# Weight Status Is Related with Gender and Sleep Duration but Not with Dietary Habits and Physical Activity in Primary School Italian Children

**DOI:** 10.3390/nu9060579

**Published:** 2017-06-06

**Authors:** Alice Rosi, Maria Vittoria Calestani, Liborio Parrino, Giulia Milioli, Luigi Palla, Elio Volta, Furio Brighenti, Francesca Scazzina

**Affiliations:** 1 Human Nutrition Unit, Department of Food and Drug, University of Parma, 43125 Parma, Italy; alice.rosi.g@gmail.com (A.R.); vittoria.calestani@hotmail.com (M.V.C.); furio.brighenti@unipr.it (F.B.); 2 Sleep Disorders Center, Department of Medicine and Surgery, University of Parma, 43126 Parma, Italy; liborio.parrino@unipr.it (L.P.); giulia.milioli@gmail.com (G.M.); 3 Department of Medical Statistics, Faculty of Epidemiology and Population Health, London School of Hygiene and Tropical Medicine, London WC1E 7HT, UK; luigi.palla@lshtm.ac.uk; 4 Giocampus Steering Committee, 43124 Parma, Italy; voltaelio@giocampus.it

**Keywords:** Mediterranean diet, KIDMED, sleep, physical activity, children, school, BMI, lifestyle, Giocampus

## Abstract

The prevalence of overweight and obesity in children has risen greatly worldwide. Diet and poor physical activity are the two risk factors usually examined, but epidemiological evidence exists suggesting a link between sleep duration and overweight/obesity in children. The aim of this study was to describe the relationship among body mass index (BMI), diet quality, physical activity level, and sleep duration in 690 children attending the 5th grade in primary schools (9–11 years old) in the city of Parma (Italy) involved in the Giocampus educational program. This was achieved through (i) measuring anthropometric data to compute body mass index; (ii) administering a food questionnaire to evaluate adherence to the Mediterranean Diet (KIDMED score); and (iii) administering a lifestyle questionnaire to classify children physical activity level (PAL), sleep duration, and school achievement. A highly significant negative association was found between BMI and sleep hours. Moreover, there was a significant positive association between PAL and KIDMED scores. No evidence was found of association between BMI and PAL, nor between BMI and KIDMED score. Data from this study established that BMI is correlated to gender and sleep duration, defining sleep habits as one of the factors linked to overweight and obesity.

## 1. Introduction

The incidence of overweight and obesity is increasing in all age groups worldwide. In Italy, overweight or obesity prevalence in children reach 35% in some regions, an extremely high level considering that obesity is high risk exposure for future health conditions [1,2]. In particular, overweight/obesity during childhood and adolescence is associated to a higher risk of developing chronic diseases during adulthood, such as several types of cancer, cardiovascular diseases, and metabolic syndrome [3]. The main determinants of overweight and obesity during childhood are an excessive energy intake, the lack of physical activity, and an inadequate sleep duration [4]. In the last few years, the time spent by children in outdoor activities or sports has considerably decreased, while time spent in screen activities, such as watching TV, playing videogames, or using electronic devices in their spare time has increased [5]. Even dietary habits have radically changed in recent years. The consumption of high energy density and processed foods has risen significantly at the expense of fruit, vegetables, legumes, and whole cereals, the latter being those food groups the Mediterranean diet is based upon [6]. This fact is even more alarming as a high adherence to the Mediterranean diet has been related to a lower risk of developing chronic diseases and mortality in adults [7]. Different studies have also shown that a short sleep duration is often related to a non-balanced food intake and to unhealthy lifestyle habits, such as a reduction of physical activity [8,9,10]. Nevertheless, only a few studies have analyzed the relationship among dietary habits, physical activity, and sleep duration in school-aged children [11].

For all these reasons, it is paramount to create a surveillance system for monitoring the actual incidence of overweight and obesity in children and for defining the relative contribution of lifestyle habits (dietary, physical activity and sleep related). Moreover, it is important to educate children about a healthy lifestyle, explaining the importance of a balanced diet, regular physical activity, and adequate sleep duration [12]. In this framework, schools seem to be the optimal context for promoting programs aimed at improving lifestyle habits in children [13].

In the state primary schools in the city of Parma (Italy), an educational school program named “Giocampus” has been created for improving the wellbeing of future generations through healthy eating education and promotion of physical activity. An integrated “learning through playing” approach for delivering nutritional education has been successful in improving children’s knowledge about healthy foods and a healthy lifestyle [14]. Professionally guided programs of physical education may also lead to significant progress in the development of conditional and coordinative abilities [15].

The aim of this study was to describe the relationship among body mass index (BMI), adherence to the Mediterranean diet (MD), physical activity level (PAL), and sleep duration in school-aged children attending the Giocampus program.

## 2. Materials and Methods 

### 2.1. Participants and Study Design

The study was carried out during the 2015–2016 school years in the city of Parma (North Italy), in primary schools participating to the Giocampus program. All the students enrolled in the fifth grade (9–11 years old) were asked to participate in this observational study, through letters sent to the schools. Before acceptance, school principals, teachers, and parents were fully informed about the study protocol and the methods of assessment. 

Data for each child were collected on the same day during school hours by two trained researchers through (i) measuring anthropometric data; (ii) administering a diet questionnaire aimed at defining the adherence to the MD; and (iii) administering a lifestyle questionnaire allowing classification of children PAL, sleep duration, and school achievement. The two questionnaires were administered directly to children.

The study was performed according to the Declaration of Helsinki and was approved by the Ethical Committee of the University of Parma (n5348-15/02/16).

### 2.2. Anthropometric Measurements

Anthropometric measurements were collected in the morning, during the physical activity class, ensuring privacy for each child, and following the WHO guidelines [16]. Body weight was measured to the nearest 100 g by using an electronic scale (MQ919, Maniquick, Niederkassel, Germany) with the child wearing only T-shirt and shorts, and was then corrected according to a simplified method validated within the Italian national surveillance system Okkio alla SALUTE [17]. Height was measured to the nearest 100 mm using a portable stadiometer (Leicester Tanita HR 001, Tanita, IL, USA). BMI was calculated as weight in kilograms divided by the square of the height in meters. Weight status was defined through the International Obesity Task Force gender- and age-related cut-offs for children BMI [18]. 

### 2.3. Dietary Habits 

Adherence to the MD was assessed through the Mediterranean Diet Quality Index for children and adolescents (KIDMED) [19]. The KIDMED questionnaire comprises 16 dichotomous yes/no questions related to 12 positive and 4 negative dietary habits. Based on their correspondence with the principles of the MD, questions with a positive connotation were scored +1 point and questions with a negative connotation −1 point. A total KIDMED score ranging from 0 to 12 points was calculated for each child. The adherence to the MD was considered low, medium, or high if the KIDMED score was ≤3, between 4 and 7, and ≥8, respectively. 

### 2.4. Physical Activity Level 

According to the Italian national survey on children lifestyle [20], the PAL of children was defined by asking children about four types of activity they may be practicing during a usual week: transport-related activity, leisure time, screen-related activity, and sport. For each question, children chose one out of four possible answers, to which a score between 1 (sedentary habit) and 4 (high physical activity) was assigned. The mean score of the questions corresponded to the final activity level of the child, in keeping with the PAQ-C questionnaire [21]. Based on their final score, children were classified into one of four PAL categories as sedentary, low active, active, and very active [22,23].

### 2.5. Sleep Behaviors

Sleep habits were explored in terms of sleep duration and sleep pattern by asking about the wake up time in the morning and the time children went to sleep, for both weekdays and weekend days. Total sleep time was calculated in hours as the difference between bedtime and wake up time for weekdays and weekend days, and as the average weighted duration using the equation: (weekday time × 5 + weekend day time × 2)/7.

On the basis of the National Sleep Foundation recommendations for school-age children [24], sleep duration was classified as low if less than 9 h per night, recommended if between 9 and 11 h per night or high if more than 11 h per night.

In addition, sleep pattern was defined using the median value of the total week average sleep-wake schedule and classified as early bed/early rise (EE) (before 22:04 and before 07:38), early bed/late rise (EL) (before 22:04 and after 07:38), late bed/early rise (LE) (after 22:04 and before 07:38), or late bed/late rise (LL) (after 22:04 and after 07:38) [25,26]. 

### 2.6. School Achievement

School performance was assessed by asking the average grade (across subjects) of the current school year as a number, since in Italy school grades could range between 0 (very poor) and 10 (outstanding). In addition, child response was classified in three categories: mostly 10 and 9 (excellent-very good level), mostly 8 and 7 (good-very satisfactory level), and mostly 6 or less (satisfactory-poor level). 

### 2.7. Statistical Analysis

All data were analyzed using descriptive statistics. Continuous variables were expressed as mean ± standard deviation (SD) of the total samples and by gender groups, BMI groups, or adherence to the MD groups. Categorical variables are presented as absolute frequencies and percentages of the total in the sample of respondents and by BMI groups or adherence to the MD groups.

The Kolmogorov–Smirnov test was applied to assess the normality of data distribution. The Student *t*-test was used to compare continuous variables between gender or BMI groups, while one-way ANOVA with Bonferroni post hoc test was used to compare among adherence to the MD groups, once the equality of variance was assessed by using the Levene’s test. A Pearson chi-square test was performed to compare categorical variables between genders, BMI groups, and adherence to the MD groups.

The statistical analysis was completed through the Statistical Package for the Social Sciences (SPSS^®^, version 24.0, IBM, Chicago, IL, USA), with the significance set at *p* < 0.05.

## 3. Results

From a total of 1062 potentially eligible children, written consent to participate from parents was collected for 711 students (response rate 67%). In addition, 21 pupils did not give their verbal consent to participate or were absent during the assessment day. A total of 690 children, 357 females (52%) and 333 males (48%), with a mean age of 10.8 ± 0.4 years old, correctly completed all study requests. Children characteristics for the total sample and by gender are presented in Table 1.

No differences were found for weight and height between genders. The mean BMI corresponded to a normal weight status defined through the IOTF gender- and age-related cut-offs for both genders, despite its being higher in males (*p* = 0.019). In general, children had a medium adherence to the MD even by gender, with females being more adherent to the principles of the MD (*p* = 0.034). On the other hand, gender frequencies appear to differ by PAL (χ^2^ = 17.1, *df* = 3, *p* < 0.001), which was representative of a medium/high active lifestyle for both genders. According to the school-age children recommendations, children slept the recommended hours per night (9–11 h), and the sleep duration was found to be higher in females (*p* = 0.010 for the average total sleep and *p* < 0.001 for both weekdays and weekend days). School achievement was similar between genders, showing a good school performance of participants. 

Irrespective of gender, 500 children (72.5%) had a low or normal weight, while 190 children were overweight-obese (Table 2). The two BMI groups frequencies appear to differ by gender (χ^2^ = 8.7, *df* = 1, *p* = 0.003), sleep duration (χ^2^ = 9.7, *df* = 2, *p* = 0.008), and school achievement categories (χ^2^ = 12.0, *df* = 2, *p* = 0.002), while they were similar for adherence to the MD, physical activity level, and sleep pattern. In addition, children in the under-normal weight group slept on average more hours per night (*p* = 0.005), and similar results were observed considering only weekdays or weekend days (*p* = 0.017 and *p* = 0.033, respectively). 

In relation to dietary habits, as shown in Table 3, only 9% of children showed a low adherence to the MD, while 55% showed a medium adherence, and 36% a high adherence. Associations were observed between adherence to MD and gender (χ^2^ = 8.5, *df* = 2, *p* = 0.015), MD and physical activity level (χ^2^ = 23.3, *df* = 6, *p* = 0.001), MD and school achievement categories (χ^2^ = 10.9, *df* = 4, *p* = 0.028). Children with a low adherence to the MD had also a lower mean sleep duration (*p* = 0.010) and a lower sleep time during weekdays (*p* = 0.002). 

**Figure 1:**
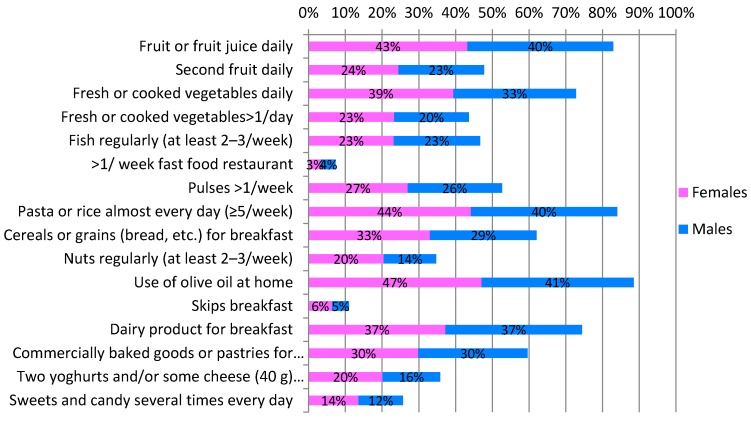
Responses to the KIDMED questionnaire of all children and by gender (percentage in respect to the total sample).

Consistently with their adherence to the MD, children showed positive eating habits, with only small variations registered by gender. The KIDMED questionnaire responses to each single question for the total sample and by gender are presented in Figure 1. 

Fruit and fruit juices were consumed daily by 83% of children, 48% had a second portion every day, and 35% ate nuts regularly. In addition, 73% of children had fresh or cooked vegetables regularly once a day, and 44% ate more than one portion of vegetables each day. In relation to protein-based food, 47% of participants ate fish regularly and 53% had pulses more than once a week. Pasta or rice was consumed almost every day by 84% of children. Almost 90% of children had breakfast regularly, 62% consumed cereals or grains for breakfast, 74% milk or dairy products, and 60% commercially baked goods or pastries. In addition, 36% ate two yoghurts and/or some cheese daily, and 88% used olive oil as a condiment when eating at home. Considering unhealthy habits, only 7% of students went more than once per week to a fast food restaurant, and 26% had sweets and/or candy several times every day.

## 4. Discussion

This study describes the relationship among body mass index, diet quality, physical activity level, and sleep duration in a sample of healthy children (aged 9–11 years) attending an educational school program in Parma, Italy.

The amount of children classified as overweight or obese was 28%, a slightly lower percentage than the value (29%) observed in the region, where the city of Parma is located (Emilia-Romagna) [20]. In turn, the regional prevalence of overweight and obesity is lower when compared to the national situation (31%) [20]. The present study involved about 700 children and the prevalence of overweight and obesity is confirmed to be lower that observed in the data from another study, with a very similar sample size, conducted in Italy [27]. Our results showed a significantly higher overweight and obesity prevalence in boys than in girls, which is consistent with recent investigations [1]. 

In our study, children showed a medium adherence to the MD, with females significantly more faithful to the principles of the MD, showing a higher attention towards healthy behaviors. However, among 33 studies analyzing gender differences in MD adherence, only 7 studies were able to significantly spot it, and 6 out of 7 reported a higher adherence in girls [28]. In the studied population, the mean MD score (6.5) was higher than that observed in the most recent studies carried out in Italy [28]. Ferranti and colleagues [25] found a mean MD adherence of 4.3 in a population of more than 1500 adolescents. In another study, conducted on a Sicilian adolescent population, the mean MD score was 5.8 in rural areas and 4.8 in urban areas [29].

The detailed analysis obtained from each single question of the KIDMED questionnaire showed positive dietary behaviors for breakfast, fruit, vegetable, and olive oil consumption. However, some other aspects can still be improved through specific educational interventions, targeted for example to promoting fish, pulse, and nuts consumption, the intake of which is lower than the national recommendations, consistently with results from other Italian surveys [20,27]. No evidence was found of an association between BMI and MD score, in partial agreement with other studies. Actually, only 10 out of 26 papers published on the topic reported an inverse association of MD adherence with BMI values [28].

A significant association was observed between MD scores and PAL. This result is aligned with the findings of 14 out of 17 studies found in the literature and investigating the association of MD adherence with lifestyle aspects in children and adolescents [28]. Our study population showed a medium-high active lifestyle with only 13% classified as low PAL. This last result is slightly better than the national data [20], reporting 16% of “non-active” children. This may be partially due to the Giocampus experience, during which children received, each school year, a 2-h/week professionally guided program of physical activity that was found to improve their motor abilities [15], and may increase children’s sensibility towards active lifestyles. In agreement with national data [20], a significantly more active lifestyle was observed for males.

No evidence was found of any association between BMI and PAL. In contrast, a highly significant negative association was found between BMI and sleep hours, although more than 76% of children declared sleeping the recommended amount of hours per night. Moreover, children with a lower sleep duration were also characterized by a low adherence to the MD, while children with a medium or high adherence to the MD slept for a similar number of hours, considering both mean and weekdays sleep quantity. These finding are aligned with results from the study by Ferranti and colleagues [25], where shorter sleep durations and poor sleep were associated with higher BMI and with unhealthy eating behaviors in adolescents. The results of this study are in agreement with the national survey “Okkio alla salute” [20], where the prevalence of overweight and obesity is significantly higher in children who sleep less hours per night. Moreover, results from the Quebec Longitudinal Study in preadolescents showed that, for each hour less of sleep per night at 10 years of age, a child was 1.5 times more likely of being overweight or 2.1 times of being obese at 13 years of age [30]. Adolescents with a longer wakefulness had a higher intake of high density snacks [31]. Moreover, results from a longitudinal study in the United States highlight bedtime as a potential target for weight management during adolescence and during the transition to adulthood [32]. In general, the importance of sleep patterns in the lifestyle of children is accepted worldwide, despite obvious cultural differences among countries [31].

In our study, we did not find any association between sleep patterns and overweight/obesity in agreement with Ferranti and colleagues [25]. However, He and colleagues [32] found that a high habitual variability in sleep patterns, but not the habitual sleep duration, was related to increased energy and food intake in adolescents, suggesting that the maintenance of a regular sleep pattern may decrease the risk of obesity.

Finally, both high adherence to the MD and normal body weight status seem to be correlated to high school achievement, suggesting a link between healthy lifestyle and academic performance. Recently, Tonetti and colleagues [33] found that a higher BMI was associated with a poorer school performance in Italian high school students when controlling for sleep quality, sleep duration, and socioeconomic status. 

The main limitations of this study are linked to the biases inherent in the use of self-reported data, such as dietary habits and physical activity level. Besides, non-response bias shall also be considered a limitation due to the fact that the sample of respondents might not be representative of the entire potential population, and the lack of information on the non-response children (33%) could not allow bias corrections. Another limitation is that children lifestyle behaviors are affected by parental habits, which were not investigated in the present study.

## 5. Conclusions

In conclusion, this study investigated lifestyle behaviors of a sample of primary school Italian children who were enrolled in the fifth and last year of the Giocampus school program, in Parma, Italy. The prevalence of overweight and obesity was lower in this group when compared to both the regional and national situations. In addition, the adherence to the MD and the PAL were higher than the ones reported in nationwide surveys, suggesting that the Giocampus school programme may represent a relevant contribution to the attainment of healthy lifestyles in children. Additionally, the high/very high mean amount of sleep hours highlighted a healthy lifeslyle pattern and, interestingly, was negatively and significantly associated with body weight. 

Based on these observations, the prevention of child obesity requires a multidisciplinary approach that considers not only physical activity and a healthy diet but also great care in defining sleeping habits. Promoting virtuous sleep behaviors may represent an important and relatively low-cost strategy for reducing the incidence of childhood obesity.

Further studies are recommended to better understand the role of sleep duration and sleep related behaviors in child energy balance. 

## Figures and Tables

**Table 1:** Participant characteristics (total sample and by gender).

Characteristic	Total Sample (*n* = 690)	Female (*n* = 357)	Male (*n* = 333)	*p* Value
Age (years)	10.8 ± 0.4	10.8 ± 0.3	10.8 ± 0.4	0.175
Weight (kg)	39.6 ± 8.7	39.2 ± 8.6	40.1 ± 8.8	0.152
Height (cm)	144.7 ± 6.8	145.0 ± 7.2	144.5 ± 6.5	0.357
BMI (kg/cm^2^)	18.8 ± 3.2	18.5 ± 3.1	19.1 ± 3.3	0.019
KIDMED Score	6.5 ± 2.2	6.7 ± 2.1	6.4 ± 2.3	0.034
Physical Activity Level				<0.001
Low	92 (13.3)	57 (8.3)	35 (5.1)	
Medium	129 (18.7)	82 (11.9)	47 (6.8)	
High	372 (53.9)	177 (25.7)	195 (28.3)	
Very high	97 (14.1)	41 (5.9)	56 (8.1)	
*Sleep Duration*				
Total Sleep (hh)	9.5 ± 0.8	9.6 ± 0.7	9.4 ± 0.8	0.010
Week days (hh)	9.3 ± 0.8	9.4 ± 0.7	9.2 ± 0.8	<0.001
Weekend days (hh)	10.0 ± 1.4	10.3 ± 1.3	9.7 ± 1.5	<0.001
School Achievement	8.5 ± 1.0	8.5 ± 1.0	8.4 ± 1.0	0.467

Data are presented as mean ± SD of 690 (total sample), 357 (female) and 333 (male) independent measurements or as frequency (% of the total sample). A Pearson chi-square test was used to test the association of physical activity level with gender, while a *t*-test was used to compare all the other variables by gender.

**Table 2:** Dietary habits, lifestyle, school achievement, and sleeping behaviors by BMI.

Variable	Total Sample (*n* = 690)	Under-Normal Weight (*n* = 500)	Overweight—Obese (*n* = 190)	*p* Value
*Gender*				0.003
Female	357 (51.7)	276 (40.0)	81 (11.7)	
Male	333 (48.3)	224 (32.5)	109 (15.8)	
*KIDMED Score*				0.881
Low	64 (9.3)	46 (6.7)	18 (2.6)	
Medium	381 (55.2)	279 (40.4)	102 (14.8)	
High	245 (35.5)	175 (25.4)	70 (10.1)	
*Physical Activity Level*				0.729
Low	92 (13.3)	66 (9.6)	26 (3.8)	
Medium	129 (18.7)	96 (13.9)	33 (4.8)	
High	372 (53.9)	272 (39.4)	100 (14.5)	
Very high	97 (14.1)	66 (9.6)	31 (4.5)	
*Sleep Duration*				0.008
Low	149 (21.6)	94 (13.6)	55 (8.0)	
Recommended	525 (76.1)	392 (56.8)	133 (19.3)	
High	16 (2.3)	14 (2.0)	2 (0.3)	
*Sleep Pattern*				0.518
EE	211 (30.6)	149 (21.6)	62 (9.0)	
EL	123 (17.8)	93 (13.5)	30 (4.3)	
LE	125 (18.1)	86 (12.5)	39 (5.7)	
LL	231 (33.5)	172 (24.9)	59 (8.6)	
*Sleep Time Quantity*				
Mean sleep (hh)	9.5 ± 0.8	9.6 ± 0.8	9.4 ± 0.8	0.005
Week days (hh)	9.3 ± 0.8	9.4 ± 0.8	9.2 ± 0.8	0.017
Weekend days (hh)	10.0 ± 1.4	10.1 ± 1.4	9.8 ± 1.4	0.033
*School Achievement*				0.002
Mostly 10 and 9	360 (52.2)	279 (40.4)	81 (11.7)	
Mostly 8 and 7	314 (45.5)	213 (30.9)	101 (14.6)	
Mostly 6 or less	16 (2.3)	8 (1.2)	8 (1.2)	

Data are presented as frequency (% of the total sample) or as mean ± SD out of 690 (total sample), 500 (under weight and normal weight children), and 190 (overweight and obese children) independent measurements. A Pearson chi-square test was used to test the association of all categorical variables with BMI groups, while a *t*-Test was used to compare Sleep Time Quantity between BMI groups.

**Table 3:** Dietary habits, lifestyle, school achievement, and sleeping behaviors by adherence to the Mediterranean diet (MD).

Variable	Total Sample (*n* = 690)	Low Adherence (*n* = 64)	Medium Adherence (*n* = 381)	High Adherence (*n* = 245)	*p* Value
*Gender*					0.015
Female	357 (51.7)	25 (3.6)	190 (27.5)	142 (20.6)	
Male	333 (48.3)	39 (5.7)	191 (27.7)	103 (14.9)	
*BMI category*					0.087
Underweight	51 (7.4)	4 (0.6)	27 (3.9)	20 (2.9)	
Normal weight	449 (65.1)	42 (6.1)	252 (36.5)	155 (22.5)	
Overweight	157 (22.8)	15 (2.2)	92 (13.3)	50 (7.2)	
Obese	33 (4.8)	3 (0.4)	10 (1.4)	20 (2.9)	
*Physical Activity Level*					0.001
Low	92 (13.3)	12 (1.7)	54 (7.8)	26 (3.8)	
Medium	129 (18.7)	17 (2.5)	81 (11.7)	31 (4.5)	
High	372 (53.9)	29 (4.2)	205 (29.7)	138 (20.0)	
Very high	97 (14.1)	6 (0.9)	41 (5.9)	50 (7.2)	
*Sleep Duration*					0.315
Low	149 (21.6)	19 (2.8)	81 (11.7)	49 (7.1)	
Recommended	525 (76.1)	45 (6.5)	289 (41.9)	191 (27.7)	
High	16 (2.3)	0 (0.0)	11 (1.6)	5 (0.7)	
*Sleep Pattern*					0.101
EE	211 (30.6)	17 (2.5)	111 (16.1)	83 (12.0)	
EL	123 (17.8)	6 (0.9)	65 (9.4)	52 (7.5)	
LE	125 (18.1)	15 (2.2)	69 (10.0)	41 (5.9)	
LL	231 (33.5)	26 (3.8)	136 (19.7)	69 (10.0)	
*Sleep Time Quantity*					
Mean sleep (hh)	9.5 ± 0.8	9.2 ± 0.8 ^a^	9.5 ± 0.8 ^b^	9.6 ± 0.8 ^b^	0.010
Week days (hh)	9.3 ± 0.8	9.0 ± 0.7 ^a^	9.3 ± 0.8 ^b^	9.4 ± 0.7 ^b^	0.002
Weekend days (hh)	10.0 ± 1.4	9.7 ± 1.9	10.0 ± 1.4	10.1 ± 1.3	0.182
*School Achievement*					0.028
Mostly 10 and 9	360 (52.2)	22 (3.2)	205 (29.7)	133 (19.3)	
Mostly 8 and 7	314 (45.5)	39 (5.7)	170 (24.6)	105 (15.2)	
Mostly 6 or less	16 (2.3)	3 (0.4)	6 (0.9)	7 (1.0)	

Data are presented as frequency (% of the total sample) or as mean ± SD out of 690 (total sample), 64 (children with a low adherence to the Mediterranean Diet (MD)), 381 (children with a medium adherence to the Mediterranean Diet), and 245 (children with a high adherence to the Mediterranean Diet) independent measurements. A Pearson chi-square test was used to test the association of all categorical variables with adherence to Mediterranean Diet, while an ANOVA with a Bonferroni post hoc test was used to compare Sleep Time Quantity among adherence to MD groups (“a,b”: different letters in the same raw indicate significant differences among adherence groups).
